# Before the ICU: does emergency room hyperoxia affect outcome?

**DOI:** 10.1186/s13054-018-1980-6

**Published:** 2018-03-08

**Authors:** Martin Wepler, Julien Demiselle, Peter Radermacher, Pierre Asfar, Enrico Calzia

**Affiliations:** 1 Klinik für Anästhesiologie, Universitätsklinikum Ulm, 89081 Ulm, Germany; 2 Institut für Anästhesiologische Pathophysiologie und Verfahrensentwicklung, Universitätsklinikum Ulm, Helmholtzstrasse 8/1, 89081 Ulm, Germany; 3 Médecine Intensive—Réanimation et Médecine Hyperbare, Centre Hospitalier Universitaire Angers, 49933 Angers, France

There is now ample evidence that hyperox(em)ia—that is, increased inspired oxygen concentrations (F_I_O_2_) and the subsequent rise in arterial oxygen tensions (PaO_2_)—coincides with aggravated mortality [1]. Most of the data originate from retrospective analyses, but a single-center trial showed that “conservative” PaO_2_ (70–100 mmHg) halved mortality when compared to “conventional” targets (≤ 150 mmHg) [2]. The available studies mostly refer to data from intensive care unit (ICU) patients, but despite its frequent use in daily practice, the impact of hyperox(em)ia remains much less clear for patients in the emergency department (ED) and/or even prior to hospital admission. Hyperox(em)ia is often present after initiation of mechanical ventilation, most likely for fear of hypoxemia when blood gas analyses are not readily available. However, supplemental O_2_ can also yield hyperoxemic PaO_2_ levels without mechanical ventilation: in the aforementioned clinical trial demonstrating the beneficial effect of targeting “conservative” *PaO*_*2*_ levels in the ICU, upon admission into the study only 2/3 of the patients investigated were mechanically ventilated [2]. However, the duration of mechanical ventilation per se is directly related to adverse outcome in ED patients.

Mechanical ventilation in the ED is mostly initiated upon the necessity for airway management, in particular in the unconscious patient (e.g., in the context of intoxication, metabolic crises, and/or traumatic brain injury (TBI)), respiratory failure (e.g., pneumonia and/or exacerbation of chronic obstructive pulmonary disease (COPD)), circulatory shock, and/or after cardiac arrest. Hence, the question arises: depending on the underlying conditions, does hyperox(em)ia affect the outcome of patients in the ED, in particular when they require mechanical ventilation? It is well established that hyperoxemia (defined as a PaO_2_ > 100 mmHg) is associated with adverse outcome in patients necessitating mechanical ventilation due to exacerbation of chronic lung disease (i.e., asthma or COPD) [3]. While there are no clinical studies on the impact of hyperox(em)ia in patients with community-acquired pneumonia, a recent retrospective study in this journal showed that hyperoxemia (defined as PaO_2_ > 120 mmHg) increased the risk of ventilator-associated pneumonia in patients receiving mechanical ventilation for more than 48 h [4]. The recent HYPER2S trial yielded deleterious effects of hyperoxemia in patients with septic shock (44% of pulmonary origin): F_I_O_2_ = 1.0 during the first 24 h after initial hemodynamic stabilization increased mortality at days 28 and 90 despite a significantly lower sequential organ failure assessment (SOFA) index at day 7, but without affecting the rate of secondary pneumonia or infection in general [5].

During the acute phase of circulatory shock, “the administration of oxygen should be started immediately to increase oxygen delivery and prevent pulmonary hypertension” [6]. The results of the HYPER2S trial suggest that hyperox(em)ia is deleterious in situations of distributive shock where “the main deficit lies in the periphery, with … altered oxygen extraction” [6]. What about shock characterized by low cardiac output and, hence, inadequate oxygen transport? While there are no data on the outcome effects of hyperox(em)ia in cardiogenic shock, it is well established that it increases systemic vascular resistance in patients with congestive heart failure [7]. In line with this, two large randomized, controlled trials have shown that hyperoxemia started already during the prehospital phase offers no survival benefit at all [8] and can even increase mortality [9] in patients with acute myocardial infarction, possibly to the preferential vasoconstrictor effect of oxygen in the coronary circulation [7]. In contrast, the role of hyperox(em)ia during hypovolemia, in particular due to trauma and hemorrhage, is much less clear: due to the blood loss-related drop in oxygen transport capacity, hyperox(em)ia is frequently used to restore tissue oxygen supply, because efficient repayment of the tissue oxygen debt determines outcome after traumatic hemorrhagic shock [10]. While clinical data are so far not available, the existing experimental literature from resuscitated large animal studies suggests that any putative benefit depends on the severity of hemorrhage shock and/or the underlying comorbidity [11, 12]. It must be emphasized that the limited duration of these experiments (maximum 48 h after hemorrhage) precludes any translation to long-term effects of hyperoxemia.

The role of hyperox(em)ia in TBI is even more conflicting: clearly, due to the preferential vasoconstrictor effect of hyperoxia in the cerebral circulation [7], there is increased risk of vasospasm-induced delayed cerebral ischemia, such as that demonstrated after subarachnoid hemorrhage [13]. Clinical data in TBI patients yielded completely opposing results inasmuch as hyperox(em)ia improved or aggravated neurological outcome and mortality, or had no effect at all. This debate is highlighted by two opposing conclusions previously published in this journal: while Narotam suggested that “… a high fraction of inspired oxygen in the emergency room may be justifiable until ICU admission for the placement of invasive neurocritical care monitoring systems*”* [14], Damiani et al. concluded that *“*… hyperoxia may be associated with increased mortality in patients with *[…]* traumatic brain injury*”* [15]. The optimal PaO_2_ in TBI so far remains unknown [16].

Finally, the role of hyperox(em)ia after cardiac arrest is not definitely answered either: the available large-scale retrospective analyses as well as more recent data showed that severe hyperoxemia (PaO_2_ > 300 mmHg) was associated with increased mortality [17, 18]. However, “moderate” hyperoxemia (101 ≤ PaO_2_ ≤ 299 mmHg) did not affect survival and was even associated with improved organ function at 24 h [17]. Moreover, probability of inhospital death after cardiac arrest was lowest at PaO_2_ values of 150–200 mmHg [19].

In a single-center observational study in this journal, Page et al. [20] recently not only confirmed that hyperoxemia (as defined PaO_2_ > 120 mmHg) is common in patients mechanically ventilated already in the ED (nearly 44% of the 688 patients included), but also is an independent predictor of hospital mortality. Moreover, hyperoxemia was directly related to morbidity as mirrored by less ventilator-free, ICU-free, and hospital-free days [20]. Of note, hospital mortality worsened across hyperoxemia severity subgroups (28%, 30%, and 35% with “mild” (121 ≤ PaO_2_ ≤ 200 mmHg), “moderate” (201 ≤ PaO_2_ ≤ 300 mmHg), and “severe” (PaO_2_ > 300 mmHg) hyperoxemia, respectively). Thus, Page et al. extend the available ICU data on the effects of excess hyperox(em)ia to the situation prior to the ICU (i.e., the ED) and eventually to the prehospital setting.

The current discussion on a possible threshold value for excess hyperox(em)ia raises the questions of both the optimal PaO_2_ to be targeted and the minimal PaO_2_ needed. Oxygen is crucial for mammalian adenosine triphosphate synthesis as the final electron acceptor in the respiratory chain, but an intramitochondrial PO_2_ ≈ 0.5–1 mmHg still allows for mitochondrial function. The aforementioned retrospective data unanimously show a U-shaped relation between PaO_2_ and the risk of mortality, with a sharp increase in mortality upon “hypox(em)ia” as defined by PaO_2_ < 60 mmHg and the lowest mortality at PaO_2_ ≈ 150 mmHg [1, 19]. On the other hand, oxygen is among the strongest oxidizing agents capable of damaging any biological molecule. Oxygen in excess of the metabolic needs may lead to increased formation of reactive oxygen species (ROS) with subsequent reduction in bioavailability of nitric oxide (NO) and consecutive impairment of tissue perfusion [7]. The present study by Page et al. [20] assumes particular importance in this context: careful titration of oxygen administration is mandatory already in the ED, and presumably even during prehospital management, in order to avoid both tissue hypoxia as well as oxygen toxicity resulting from excess radical production.
